# Demand Management for Optimized Energy Usage and Consumer Comfort Using Sequential Optimization

**DOI:** 10.3390/s21010130

**Published:** 2020-12-28

**Authors:** Mikhak Samadi, Javad Fattahi, Henry Schriemer, Melike Erol-Kantarci

**Affiliations:** School of Electrical Engineering and Computer Science, University of Ottawa, Ottawa, ON K1N 6N5, Canada; msama043@uottawa.ca (M.S.); Javad.Fattahi@uottawa.ca (J.F.); hschriemer@uottawa.ca (H.S.)

**Keywords:** demand management, sequential optimization, device scheduling, smart grid

## Abstract

The Energy-efficiency of demand management technologies and customer’s experience have emerged as important issues as consumers began to heavily adopt these technologies. In this context, where the electrical load imposed on the smart grid by residential users needs to be optimized, it can be better managed when customer’s comfort parameters are used, such as thermal comfort and preferred appliance usage time interval. In this paper a multi-layer architecture is proposed that uses a multi-objective optimization model at the energy consumption level to take consumer comfort and experience into consideration. The paper shows how our proposed Clustered Sequential Management (CSM) approach could improve consumer comfort via appliance use scheduling. To quantify thermal comfort, we use thermodynamic solutions for a Heating Ventilation and Air Conditioner (HVAC) system and then apply our scheduling model to find the best time slot for such thermal loads, linking consumer experience to power consumption. In addition to thermal loads, we also include non-thermal loads in the cost minimization and the enhanced consumer experience. In this hierarchal algorithm, we classified appliances by their load profile including degrees of freedom for consumer appliance prioritization. Finally, we scheduled consumption within a Time of Use (ToU) pricing model. In this model, we used Mixed Integer Linear Programming (MILP) and Linear Programming (LP) optimization for different categories with different constraints for various loads. We eliminate the customer’s inconvenience on thermal load considering ASHRAE standard, increase the satisfaction on EV optimal chagrining constrained by minimum cost and achieve the preferred usage time for the non-interruptible deferrable loads. The results show that our model is typically able to achieve cost minimization almost equal to 13% and Peak-to-Average Ratios (PAR) reduction with almost 45%.

## 1. Introduction

Residential demand management, despite the vast research efforts on the recent years and the wide literature, persists as an open issue. In particular, studies addressing the tradeoff between utility gains and user comfort are few [1,2,3]. Existing approaches aim to maintain a smooth user demand profile, that is, to prevent peaks [4]. Customer comfort has been less considered where it could be associated with appliance usage performance, delays in responding to utility demand response requests, room temperature and so forth. To address this gap, we present a demand management approach that considers customer comfort in our multi-objective optimization model.

According to the energy internet paradigm, control technologies will play a central role in the modern grid [5]. Most of the research on Demand Side Management (DSM), or in other words residential level load control, aims to reduce either the customer’s cost or the grid operators’ PAR [6]. For instance, in [7] the authors have grouped appliances into two categories, essential demand and flexible demand, and then defined a consumption order for the appliances within their cost minimization algorithm. Meanwhile, in another article [8], the authors focus on scalability and acceptability where they categorized appliances, ordered their consumptions and defined boundaries for appliance usage time slots to bound the consumption and to make the consumption diagram smooth. In [9], the authors have clustered loads based on their priorities within a Neighborhood-Area Network (NAN) with the goal to find a tradeoff between Energy Management System (EMS) processing cost and response time delay to achieve demand-supply balance. Note that in this paper, comfort is associated with improved fairness in delay and dispatch rates. The authors in [10] have focused on minimizing the household bill based on different categories of appliances and dynamic pricing tariff using Genetic Algorithm (GA) method to find the optimal operating parameters for each individual device. Finally, in [11] they have minimized the total energy cost of appliance rescheduling, treating rescheduling as an inconvenience (that is, a discomfort).

With the increasing penetration of renewable energies at the residential level, some of the previous works on demand side management have proposed appliance scheduling based on the power available from renewable resources. A hierarchical model has been proposed in [12] to maximize Distributed Energy Resource (DER) use and to reduce the load on the grid. In this model, loads are bundled and scheduled to align their consumption patterns with available renewable resources. This approach not only reduces the load on the grid but also reduces customer cost. In [13], the authors have aimed to reduce the cost while keeping the power consumption in a building under a certain threshold. For cost minimization, they shared the DER power generated between all the residents. In [14], the authors integrated a sensor network with a home energy management system and showed that energy consumption can be reduced with a system that employs communication with the users.

DSM can also utilize the predicted day-ahead load [15]. In [16], the authors have performed day-ahead scheduling using real-time pricing by predicting next-day customer demand. Their main goal is to accurately predict the load and minimize the cost of generation. In this case, the authors have increased customer satisfaction using an incentive-based model.

One of the important comfort factors at the residential level is the temperature of the living area, which corresponds to the thermal load. The thermal load can be defined for different devices, such as a HVAC system. Most of the studies in this area have tried to minimize the thermal load while maintaining the customer’s comfort. In [17], authors have scheduled HVAC energy consumption by increasing or decreasing the room’s temperature under a price consideration. Thus, customer comfort is considered as an energy cost. In several studies, authors have added other features addition to the thermal load to improve their calculations [18,19]. In [18], the main goal was to minimize the HVAC’s cost of energy and also to maintain customer comfort. In their work, the authors looked for different parameters that affect room temperature, such as the number of occupants, indoor and outdoor temperature, and customer preference. They used a nonconvex formulation to solve their problem with tradeoff between cost and thermal comfort. The work in [19] presented central demand management to control building Air Conditioner (AC) power consumption and preferred temperature. This scheme evaluated the system communication delay, outdoor temperature and other features. Meanwhile, the authors in [20] proposed a MILP based on dynamic pricing to optimize the thermal load in a smart house and to maintain customer comfort. In [21], the authors addressed HVAC system energy conservation and wastage using a machine learning approach, using Internet of Things (IoT) sensor data to establish consumer consumption patterns.

In several articles on thermal load management, the authors have taken a thermal standard as index and addressed temperature in that context. In [22], authors used the ISO standard on residence’s comfort temperature and minimized Predicted Percentage Dissatisfaction (PPD). Then, they applied a direct load control model with Particle Swarm Optimization (PSO) to reduce thermal load. Other approaches have optimized both thermal and non-thermal loads to increase systems efficiencies, as noted in [23,24].

Most of the papers in the DSM area use a mathematical model to minimize or maximize one or many objective functions with different load constraints. For instance, the authors in [25] have implemented a forecasting model to predict household renewable generation and load, then they have applied an optimization model to schedule the appliance usage profile based on increasing EV charging, reducing the total cost and maximizing the benefit of selling renewable. The linear programming approach has been used in [26] to minimize customer cost and constrain the total usage cost to be less than a specific budget. In addition, a Mixed Integer Non-linear Programming (MINP) approach has been used in [27] to control cost and appliances usage. The idea of using MILP in DSM is to find optimal time slots (integer value) for load profiles. The multi-objective MILP in [28] has been presented to control PAR, cost and schedule inconvenience using ToU tariff. In addition, several works focus on appliance management. They have categorized such loads in different categories and then apply a suitable objective function with proper constraints to schedule them [29]. In [30,31], the authors have presented a prioritized model based on the appliance categories to minimize their costs. For more clarification, we categorized the reviewed articles in Table 1.

In this paper, we present a multi-objective sequential optimization model to distribute loads over a time horizon. The loads are categorized into three clusters of load types essential, deferrable and elastic, and for that reason we name our method as Clustered Sequential Management (CSM). Our proposed approach considers a house with a Home Energy Management System (HEMS) which is able to prioritize appliances and communicate with the users. We use ToU pricing rates as the price signal. We propose MILP and LP based optimization techniques that jointly minimizes the cost and maximize thermal comfort. Our main contribution is a multi-objective model based on different types of appliances, different priorities of appliances, utility price signal vector over peak hours and considering thermal satisfaction regarding to ASHRAE standard to maintain temperature in a standard range and prevent wasting the energy for heating the house. The main contribution of this work is employing both MILP and LP optimization models to reduce customer energy bill and PAR by considering thermal comfort jointly with a prioritized appliance scheduling. We considered a variety of load categories to assess the proposed optimization methods including non-flexible (essential loads) as well as flexible loads (elastic and deferrable loads). The proposed model has been validated in helping customers to save their energy bill using a real appliances energy profiles.

The rest of the paper is organized as follows. Section 2 presents our system model and the proposed optimization models. In Section 3, we illustrate our results, and in Section 4 we present our conclusions.

## 2. System Model and Problem Formulation

**Figure 1:**
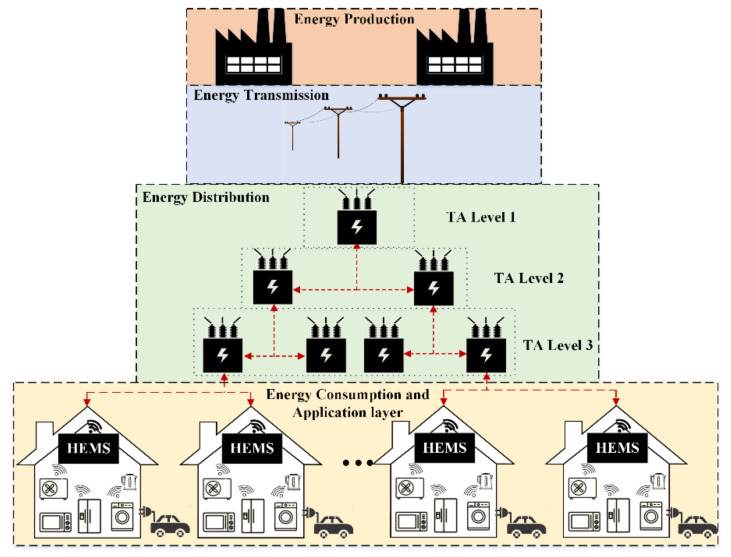
Top-down view of our IoT ecosystem.

With the advances in smart appliances, home appliances are now a part of the IoT ecosystem while the smart grid positions itself as an ideal example of an Industrial IoT (IIoT) system [32]. Figure 1, illustrates the major elements of this ecosystem. At the top level, we have generators that could be based on conventional or renewable energy sources. Then, the produced energy is transported through transmission lines to the distribution system transformers, which is called as transformer level. At energy distribution level, each Transformer Agent (TA) will balance the voltage and frequency to be suitable for residential usage by stepping up/down the voltage. At the residential level, HEMS, as an IoT device, communicates with TA to send the customer’s usage data to the utility. The household IoT devices (such as HVAC, EV, washing machine and etc.) communicate with HEMS through Wi-Fi or Zigbee and create a small network inside the house.

In this paper, we assume that the utility sends a Demand Response (DR) signal to the customers and asks them to collaborate on demand management to manage the grid supply and demand at peak times. However, customers have appliances that need to be on during certain times and they also have thermal loads that can be controlled to maintain a certain level of user satisfaction. To achieve these goals, a smart HEMS device is needed. The device is able to control and monitor the customer’s power consumption. Our aim is to minimize the cost, maximize the customer’s comfort and to reduce the PAR based on utility’s DR signal.

Let *N* be the number of customers, where i∈N is the customer index. Subscript a denotes the appliance number and $A_{i}$ is the set of appliances for customer i, where $a\in A_{i}$. The number of appliances for customer i is given by $\left|A_{i}\right|$. We subdivide the 24-hour period into T equal time slots and t∈{1,2,…,T}. An appliance profile may be defined in terms of its nominal pattern of power consumption $L_{a}=\left[l_{a}^{1},\cdots ,l_{a}^{T_{a}}\right]$, where $l_{a}^{t}$ is the appliance energy consumption in time slot *t*, and the dimensionality is expressed by the number of time slots $T_{a}$ over which the appliance operates. Its optimized operating state during the day is given by the binary vector $\tau_{a}=\left[\tau_{a}^{1},\cdots ,\tau_{a}^{T}\right]$, where the appliance condition (ON/OFF) for time slot *t* is given by $\tau_{a}^{t}$ (*i.e.*, 1 or 0). This operating state is determined by a scheduling and optimization process (described below) that transforms $L_{a}$ into $X_{a}=\left[x_{a}^{1},\cdots ,x_{a}^{T}\right]$, where $x_{a}^{t}$ is the optimized appliance consumption for time slot *t*. The customer aggregated load vector $\chi_{i}=[\chi_{i}^{1},\ldots ,\chi_{i}^{T}]$ is sequentially constructed, with $\chi_{i}^{t}$ the total optimized load for time slot *t*.

### 2.1. Load Categories and Scheduling Approach

We consider three load categories. Essential loads $\left(A_{E}\right)$ are those directly initiated by the user, lacking any HEMS control of their power consumption or profile (e.g., coffee maker). Elastic loads $\left(A_{\mathrm{El}}\right)$ are those with load profiles whose consumption may be adjusted by HEMS control within any time interval (e.g., HVAC). Such loads have a central impact on customer comfort level. Deferrable loads $\left(A_{D}\right)$ are those whose load profiles are schedulable (e.g., washing machine) within some customer-defined interval. Such loads have a central impact on customer lifestyle and convenience. For each appliance *a*, we define a binary vector $I_{a}=\left[I_{a}^{1},\cdots ,I_{a}^{T}\right]$, where

(1) $$I_{a}^{t}=\left\{\begin{array}{cc} 1 & t_{s}\leq t\leq t_{f}\\ 0 & otherwise \end{array}\right.,\;\forall I_{a}^{t}\in I_{a}$$

denotes the permissible scheduling interval in terms of starting and finishing time slots $t_{s}$ and $t_{f}$, respectively. This permits time constraints to be set. One fundamental constraint is that the permissible interval be greater than the usage time $T_{a}$, where

(2) $$\left|t_{f}-t_{s}\right|>T_{a}$$

$A_{i}$ is composed of distinct subsets and may be represented as:

(3) $$A_{i}=\left\{A_{E}{,A}_{\mathrm{El}}{,A}_{D}\right\}.$$

Appliances may also be categorized by their usage priority, with essential loads being mandatory. For all other loads, priority levels are customer-assigned via the HEMS, but elastic loads are assumed to have a higher priority than deferrable ones. The appliances priority is denoted by $\Gamma_{i}=[\rho_{1},\ldots ,\rho_{M}]$ with length of $M=\left|A_{\mathrm{El}}\right|+\left|A_{D}\right|$, whose element $\rho_{a}$ is appliance a priority coefficient. To allocate the priority coefficients to these appliances, we use the Analytic Hierarchy Process (AHP) [33] in our optimization model.

**Figure 2:**
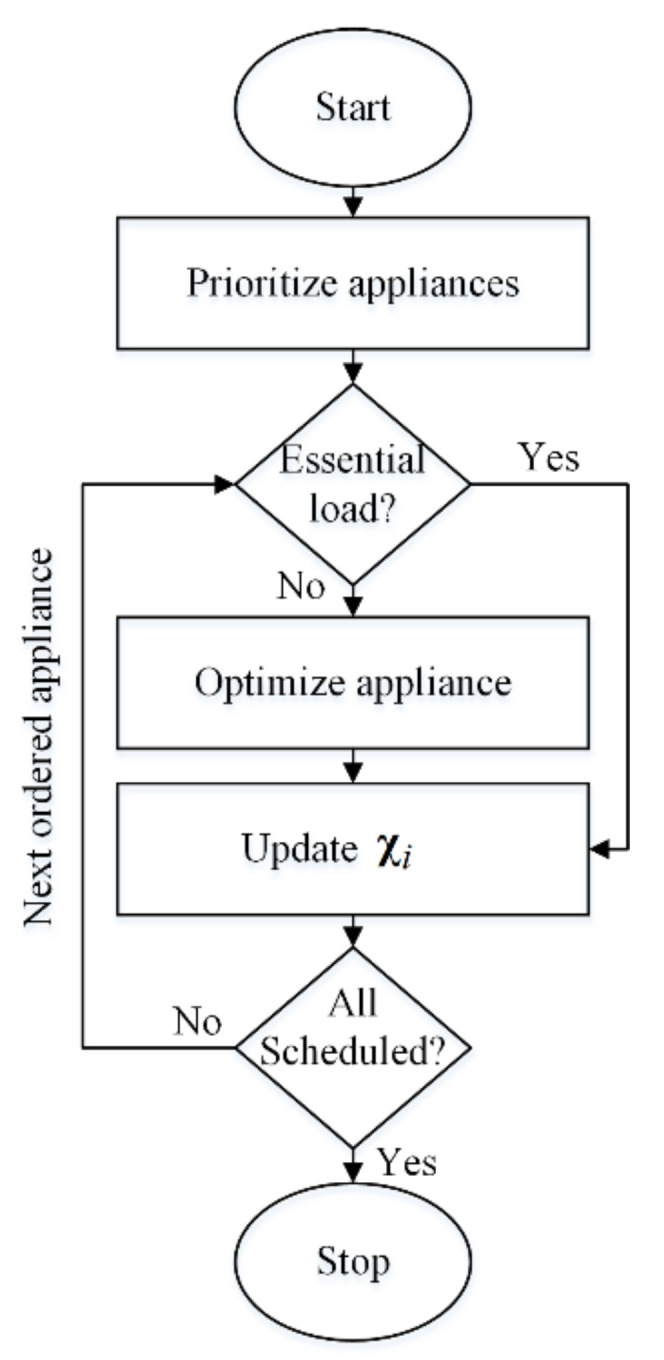
The flowchart of our proposed model.

We implement load optimization, described in the following subsection, within a sequential approach. This is illustrated in the flowchart provided in Figure 2. This sequential scheduling considers the appliance load profiles entered into the load vector by order of priority. Note that the summation of $\chi_{i}$ across the time horizon should be almost equal to the summation of all the appliances’ load profiles:

(4) $$\sum_{t=1}^{T}\chi_{i}^{t}=\left(\sum_{a=1}^{\left|A_{i}\right|}\sum_{t=1}^{T_{a}}l_{a}^{t}\right)\pm \Delta .$$

### 2.2. Optimization

The ultimate goal of the proposed optimization scheme is to minimize the total residential energy consumption which is given by $\min\;f\left(x_{a}\right)\;+\;g\left(x_{a},\tau_{a}\right)$. Each appliance is contributing to this optimization model by minimizing their own consumption as explained below. Constraints specific to appliances type, essential, elastic or deferrable, are applied. We optimize $X_{a}$ and $\tau_{a}$ by sequentially minimizing the cost of the incremented daily load,$\chi_{i}\leftarrow X_{a}+\chi_{i}$, using a general time-of-use price signal $P=\left[p^{1},\cdots ,p^{T}\right]$.

#### 2.2.1. Elastic Load Model

Elastic devices have a defined operating state $\tau_{a}$ (i.e., are not schedulable) but their power consumption $x_{a}$ is adjustable. By considering the general form of optimization, we can define an LP optimization model for this category as

(5) $$\min f\left(x_{a}\right)=\min\sum_{t=t_{s}}^{t_{f}}p^{t}\left(x_{a}^{t}+\chi_{i}^{t}\right)\;\mathrm{Subj}.\mathrm{to}\;\beta \sum_{t=t_{s}}^{t_{f}}\log\left(x_{a}^{t}+1\right)\geq S_{a}\;\;\left(1-\beta \right)\mathrm{PPD}\left(x_{a}^{t}\right)\leq D_{a},\;\;t\in \left[t_{s},t_{f}\right]\;\;\;\;\;\chi_{i}^{t}\leq x_{a}^{t}+\chi_{i}^{t}\leq Ub^{t}$$

where $p^{t}$ is ToU pricing signal; $x_{a}^{t}\in X_{a}$ is appliance a consumption for time slot *t*; $\chi_{i}^{t}\in \chi_{i}$ is the aggregated load vector of customer i at time slot *t*; and $t_{s}$ and $t_{f}$ are the appliance’s preferred starting and finishing work time intervals, respectively. If the appliance is a non-thermal elastic load, β=1(such as EV), otherwise β=0 (such as HVAC). The first constraint is specifically used for non-thermal loads, where $S_{a}$ is the minimum level of power consumption extracted as in [34]. A logarithmic function is used to ensure minimum device performance in the limit as such a function saturates [35]. The second constraint is used for the thermal system and depends on environment and appliance energy dissipation. $D_{a}$ is the threshold for Predicted Percentage Dissatisfaction (PPD) to ensure customer’s dissatisfaction remains less than a certain value. We use PPD function to measure the customer’s dissatisfaction regarding room temperature [36]. PPD is defined in the ASHRAE standard and it is governed by the parameters that establish room conditions. There is an indirect relation between PPD and power consumption using Predicted Mean Vote (PMV) [17,37]. Finally, the third constraint is used for both thermal and non-thermal loads to bound each time slot between the aggregated load $\chi_{i}^{t}$ and the maximum threshold of household usage $Ub^{t}\in \mathrm{Ub}$ at time slot *t*. The goal of defining a limitation for each time slot is to prevent peak events and distribute the customer load evenly throughout the day.

#### 2.2.2. Deferrable Load Model

In the case of deferrable load scheduling, the optimization model will manage the load profile through the permissible interval and find the minimum cost. This approach is completely different than the previous model for elastic devices. In this model, instead of LP, we are using MILP. This model helps us to first find the proper time for appliance usage and then find the optimized load profile vector for appliance a. For this optimization, as a general model we have

(6) $$\min g\left(x_{a}{,\tau }_{a}\right)\;=\;\min\sum_{t=t_{s}}^{t_{f}}p^{t}\tau_{a}^{t}\left(x_{a}^{t}+\chi_{i}^{t}\right),\;\mathrm{Subj}.\mathrm{to}\;\sum_{t=t_{s}}^{t_{f}}\tau_{a}^{t}I_{a}^{t}=T_{a}\;\sum_{\tau_{a}^{t}=1\Rightarrow k=t}^{k+T_{a}}p^{k}x_{a}^{k}\leq C_{a},\;\;t_{s}\leq t\leq t_{f}-T_{a}\;\;\;\;\chi_{i}^{t}\leq \tau_{a}^{t}\left(x_{a}^{t}+\chi_{i}^{t}\right)\leq Ub^{t}$$

where $p^{t}$ is ToU pricing signal; $x_{a}^{t}\in X_{a}$ is the appliance a consumption at time slot *t*; $\chi_{i}^{t}\in \chi_{i}$ is the aggregated load of customer i at time slot t; $\tau_{a}^{t}\in \tau_{a}$ is the operating state of appliance a (the ON/OFF condition) at time slot *t*; and $t_{s}$ and $t_{f}$ are the appliance’s starting and finishing work time intervals, respectively. The first constraint is used to find $T_{a}$, which is the number of time slots the appliance needs to complete its operation within the permissible interval $I_{a}$$\left(I_{a}^{t}\in I_{a}\right)$. The second constraint is used to require uninterrupted device operation, with $C_{a}$ the minimum operation cost for appliance a in its permissible interval; $\tau_{a}^{t}=1\Rightarrow k=t$ ensures that if and only if the optimized operating state is equal to one, then the time slot and the summation of cost for the $T_{a}$ time slots after that (from k=t to $k+T_{a}$) should be lower than or equal to the minimum cost $C_{a}$. The third constraint is used to bound each time slot between the aggregated load vector and the maximum threshold of household usage at time slot *t*.

### 2.3. Thermal Model

As mentioned in Section 2.2.1, we choose a PPD rate and with PPD and PMV functions we calculate the necessary thermal load [17]. To make a map between PPD, PMV, thermal energy and temperature, we consider the fundamentals of thermal conduction. Room size, wall quality and inside and outside temperatures have direct impact on thermal loss. From [38],

(7) $$Q_{power}=C_{room}\times \frac{d\theta_{room}}{dt}$$

is used to calculate the thermal power needed to change the room temperature $\theta_{Inside}$ to the preferred temperature $\theta_{preferred}$ at a specific rate $\left(\frac{d\theta_{room}}{dt}\right)$, where $C_{room}$ is the room thermal capacity [38]. The power leakage is determined via

(8) $$Q_{leak}=\frac{\theta_{Outside}-\theta_{Inside}}{R},$$

where $\theta_{Outside}$ is the outdoor temperature and R is the room’s thermal resistance. In our model we are using both formulations with regard to ASHRAE standard room temperature.

**Figure 3:**
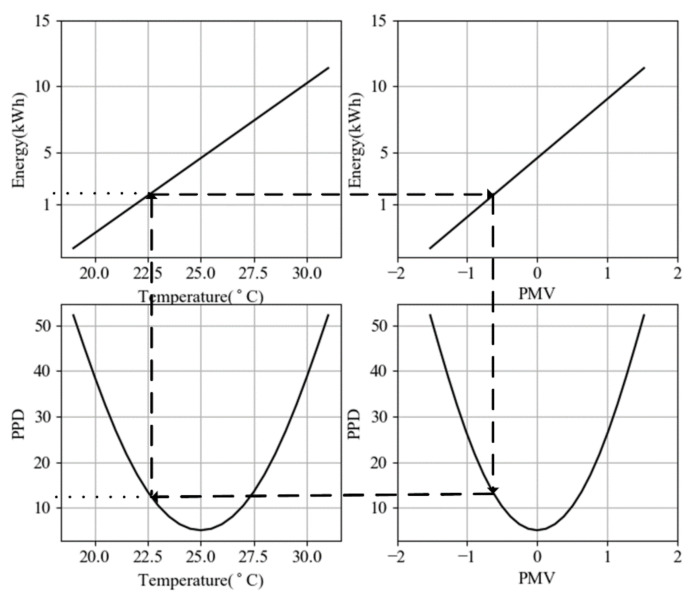
Relation between PPD, PMV, temperature and energy [Assumption:$\theta_{Outside}=-10\;{}^{\circ}C$].

Figure 3 shows that when PPD is equal to 11.68%, we need to consume almost 1.8 kWh to increase the room’s temperature from 20 ℃ to 22.5 ℃ when the outside temperature is −10 ℃. According to the ASHRAE standard [36], the optimal temperature range for a room in winter with the optimal PPD (≤10%) is between 23 ℃ and 27 ℃ which is also observable from Figure 3. Therefore, our algorithm tries to keep the temperature in this range regarding to room heating leakage, outside temperature and inside temperature.

### 2.4. Analytic Hierarchy Process (AHP)

AHP is a decision-making model that is used for ranking the alternatives when we have multi-criteria problems [33]. A pairwise comparison is made between the specified criteria and alternatives with the grades ranging from 1 to 9. The value $r\in \left\{1,\ldots ,9\right\}$ shows how much more priority an alternative have over the other. Intensity r=1 means they are equal, r=2,3 shows the moderate condition, r=4,5 means one is stronger than the other, r=6,7 one is very strong and r=8,9 presents the extreme importance of one to the other. Let’s assume we have *m* criteria and *n* alternatives then, the relative matrix $A_{k}$ for criteria *k (*$k\in \left\{1,\ldots ,m\right\})$ represents the relative rates between alternatives *i* and *j* ($\alpha_{ij}$) where $i,j\in \left\{1,\ldots ,n\right\}$ and it is calculated by $\alpha_{ij}=\frac{r_{i}}{r_{j}}$ where $r_{i},r_{j}\in \left\{1,\ldots ,9\right\}$.

(9) $$A_{k}=\left[\begin{array}{ccc} \alpha_{11} & \alpha_{12} & \begin{array}{cc} \ldots & \alpha_{1n} \end{array}\\ \alpha_{21} & \alpha_{22} & \begin{array}{cc} \ldots & \alpha_{2n} \end{array}\\ \begin{array}{c} \vdots \\ \alpha_{n1} \end{array} & \begin{array}{c} \vdots \\ \alpha_{n2} \end{array} & \begin{array}{c} \begin{array}{cc} \ddots & \vdots \end{array}\\ \begin{array}{cc} \ldots & \alpha_{nn} \end{array} \end{array} \end{array}\right]=\left[\begin{array}{ccc} 1 & \frac{r_{1}}{r_{2}} & \begin{array}{cc} \ldots & \frac{r_{1}}{r_{n}} \end{array}\\ \frac{r_{2}}{r_{1}} & 1 & \begin{array}{cc} \ldots & \frac{r_{2}}{r_{n}} \end{array}\\ \begin{array}{c} \vdots \\ \frac{r_{n}}{r_{1}} \end{array} & \begin{array}{c} \vdots \\ \frac{r_{n}}{r_{2}} \end{array} & \begin{array}{c} \begin{array}{cc} \ddots & \vdots \end{array}\\ \begin{array}{cc} \ldots & 1 \end{array} \end{array} \end{array}\right]$$

After filling the matrix, we normalize each relative rate $\alpha_{ij}$ using $\alpha_{ij}=\frac{\alpha_{ij}}{\sum_{i=1}^{n}\alpha_{ij}}$ and to calculate the alternative *i*’s weight in criteria *k*, we have $w_{i}^{k}=\frac{\sum_{j=1}^{n}\alpha_{ij}}{n}.$ Then, we extend matrix $A_{k}$ for other criteria and calculates $\;w_{i}^{k}\;,\;\;\forall \;i\in \left\{1,\ldots ,n\right\},\;k\in \left\{1,\ldots ,m\right\}$. After that, we rate the criteria relatively in the same way and multiply the criteria weight $w^{k}$ with each alternative weight $w_{i}^{k}$ and finally the alternative *i*’s priority will be calculated using $\rho_{i}=\overset{m}{\underset{k=1}{\sum }}(w^{k}\times w_{i}^{k})$.

In our model, we have implemented a two-level AHP to fairly prioritize the appliances in our sequential optimization model. We have two criteria (m=2), customer preferences on appliance usage and appliance total consumption, and 6 deferrable and elastic loads as alternatives (n=6). There might be other criteria and alternatives, but in our case we found that these are the most important ones that affect appliances scheduling. In our model, the AHP algorithm is implemented in HEMS. Then, each customer can interact with HESM and rate each two appliances relatively. Note that HEMS has the total consumption information of connected appliances. Finally, HEMS does the AHP computation and find the appliances weight or priority values.

## 3. Simulation Results

In this section, we present our simulation results and compare our model, Clustered Sequential Management (CSM), with four other demand management approaches; Multi-class Appliances Scheduling (MAS) [34], Autonomous Demand-side Management (ADM) [39], Household Energy Management (HEM) [10], and Multi-objective Household appliance Optimization (MHO) [28]. To make our implementation close to real world conditions, we use the dataset of household appliances load profile from [40]. Table 2 presents the appliances’ type and their total power consumption in a day.

The simulation environment is Python and we use SciPy library to solve MILP and LP optimization models. This simulation is conducted on Intel i5 CPU with 3.55 GHz clock speed and 16 GB RAM. Also, our algorithm processing time was 10 seconds. Four different scenarios with different mixes of appliances are used for performance evaluation. These are indicated in Table 3 and are comprised of (i) 6 essential, 2 elastic (EV and Heater) and 1 deferrable loads, (ii) 5 essential, 2 elastic (EV and Heater) and 2 deferrable loads, (iii) 4 essential, 1 elastic (EV) and 3 deferrable loads, and (iv) 3 essential, 1 elastic (Heater) and 4 deferrable loads. These are defined to compare the sensitivity and effectiveness of five different approaches (CSM, MAS, ADM, HEM and MHO) with respect to load types. Note that other combination of loads do not impact the workings of the proposed scheme. Therefore we choose these four different scenarios to evaluate the performance of our model. In MAS and ADM, all the power consumption is accumulated and distributed through the permissible intervals without considering the appliances’ priority on power consumption and customer’s preferences. However in MAS, the authors categorize the appliances into different load clusters and optimize each using their specific optimization function. Moreover, in MAS deferrable loads are non-interruptible. Also, we compare our model with other recent articles HEM and MHO. They have some similarities with our model in comfort, cost minimization and appliance scheduling. Besides these similarities, there are some differences. In HEM [10], the authors have implemented an iterating GA and assumed different load categories with different settings to adjust appliance time usage and comfort level. However the loads are optimized simultaneously without considering the essential load effects on peak and cost. On the other hand, in MHO [28], their multi-objective model focused on minimizing cost, peak, and scheduling inconvenient. The authors determined different orders of these three factors and again optimize all the appliances simultaneously with many constraints. In [28] the effect of the essential loads on the peak consumption and cost has not been considered.

To put the appliances in order for our sequential optimization, or in another words, to prioritize them, we used the AHP method which is explained in Section 2.4. This yielded the priority vector $\Gamma_{i}=[\rho_{1},\ldots ,\rho_{M}]$ for M deferrable and elastic devices. Note that in this model, elastic loads have higher priority than deferrable ones because their total consumption is higher than deferrable loads.

In this simulation, scheduling is performed across a 24-h day subdivided into 96 equal time slots beginning at 5 AM. We use a ToU pricing signal based on the Ontario Energy Board (OEB) [41], with household energy consumption based on an average winter consumption in Ontario, Canada. We assume the customer wants to keep the room temperature within the maximum permissible ASHRAE standard range and we include provisioning for fully charging an EV. We consider a room size of 118.4 square feet, with $\theta_{Outside}=-10\;{}^{\circ}C$ (the average outside temperature in December 2018 in Ontario), and an inside temperature of $\theta_{Inside}=22\;{}^{\circ}C$. We assume a PPD of less than 16%.

**Figure 4:**
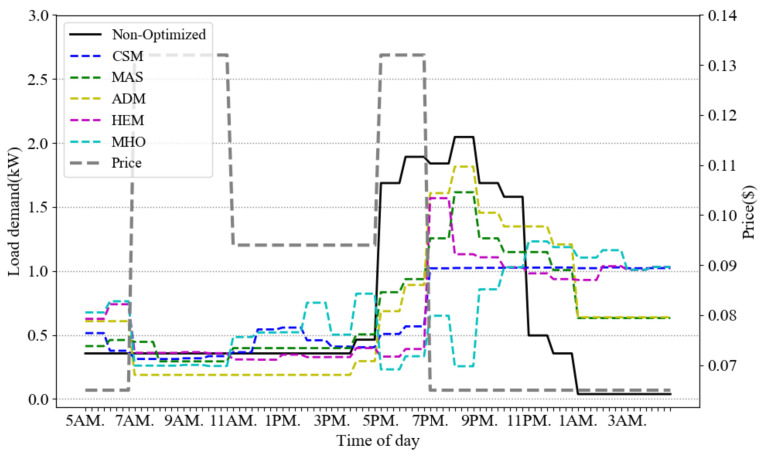
ToU rate and average energy consumption scheduling in a day of scenario A.

Figure 4 presents the optimized power consumption of scenario A in six different models: our CSM, versus MAS, ADM, HEM, MHO and the non-optimized case. Note that for MHO implementation, we choose the order of inconvenient, cost and peak optimization (scenario 3 in [28]) which is closer to our proposed architecture. This figure shows the average result of 10 runs. The price signal presents different tiers of ToU pricing (off-peak, mid-peak and on-peak). As observed from the figure, the proposed model reduces the peak consumption almost 30% more than the MAS, ADM and HEM, and 15% more than MHO in scenario A.

**Figure 5:**
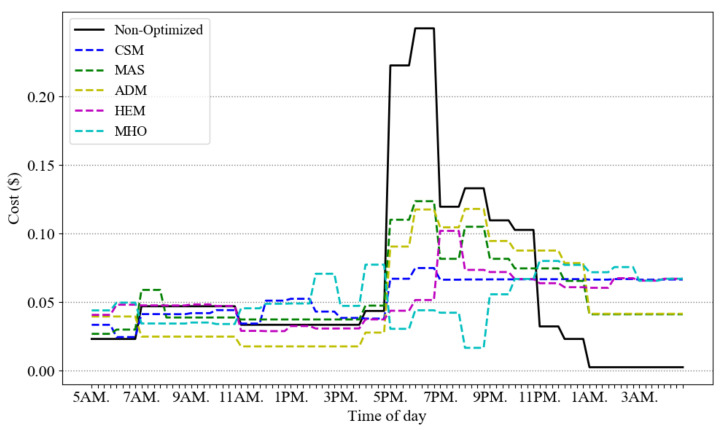
Cost changes in different time slots for five models.

**Figure 6:**
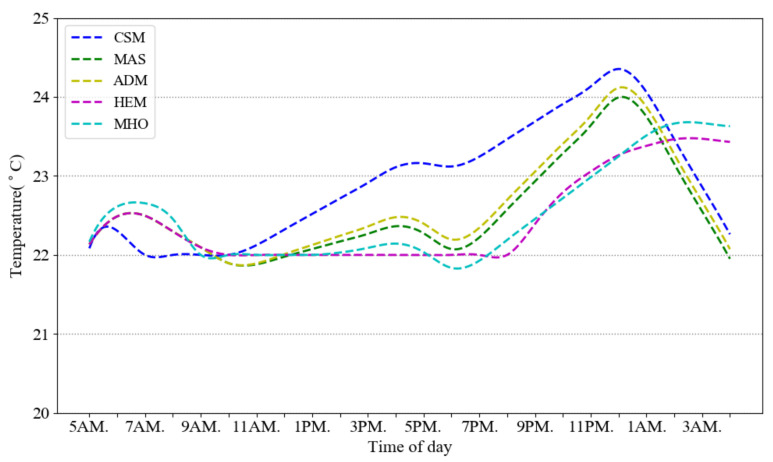
Temperature fluctuation in different models.

Figure 5 gives the cost profiles for the load demands of Figure 4. Due to the flattening impact of our CSM scheme, its overall cost is lower than the other schemes. Figure 6 illustrates how the temperature is fluctuating over different time slots in the compared approaches. The five models are consuming the same amount of power in a day to keep the room warm but their temperature is different on different time slots. Our approach is keeping the temperature in ASHRAE standard range and increasing the temperature close to 25 ℃ which is the best room temperature in winter. The approaches MAS, ADM and MHO schedule the total energy regardless of thermal comfort formulation but consuming the same minimum range of electricity for thermal load during a day.

But HEM model has a thermal constraint for setting the minimum and maximum room temperature. Here we set it between 22 ℃ and 25 ℃ same as our model assumption. CSM and HEM keeps the temperature more than 22 ℃ but our model increases the temperature more (close to 24.35 ℃) to reduce the PPD. Table 4 is a summary of the minimum and maximum temperatures and the averaged PPD in a day for the different approaches. Our CSM approach has the lowest PPD and though it does have a slightly greater temperature excursion than the other approaches, while still remaining within the limits, the rate of temperature variation is much less. HEM has higher PPD than MHO despite of having temperature constraint. The HEM guarantees to keep the temperature in the comfort range (more than  22 ℃) and minimize the bill. Therefore, at peak times it consumes the minimum electricity which is needed to satisfy the temperature constraint. But MHO is fluctuating through the times and cooling and warming the house based on the ToU pricing signal.

As a consequence, the best way for simulating a household thermal comfort is to use a standard satisfaction formulation such as PPD in optimization constraint instead of only considering the temperature range.

**Figure 7:**
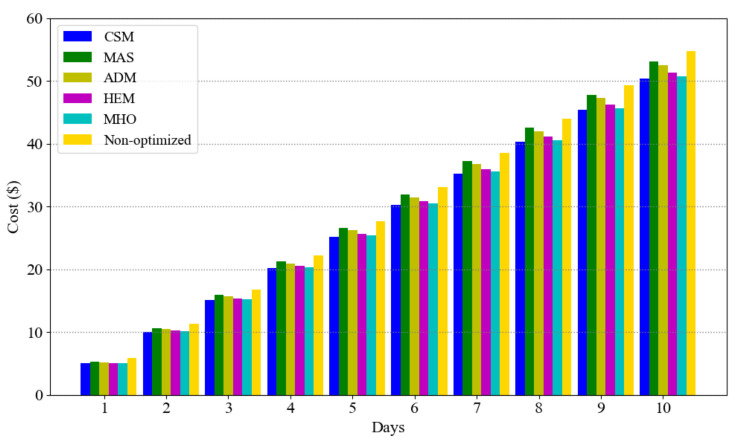
Cumulative cost in 10 days.

To ensure that our approach is robust with regard to parameter choice, we repeat scenario A for 10 days and calculate the cumulative cost for different approaches; the results are presented in Figure 7. Our approach is seen to always have less cost than the others. The reason that MAS has higher cost than ADM is that, in the former, the deferrable loads are non-interruptible which constrains usage time but in the latter they are interruptible and unconstrained. Also, HEM and MHO have almost more accumulated cost than CSM which is due to the lack of essential load consideration on their scheduling. Moreover, we can assert that within 10 days of consumption, customer saves almost $5 and if we extend it to a month the saving would be $15. Note that the average cost of electricity bill in Ontario, Canada is $125 per month [41]. Therefore, the customer’s savings would be considerable.

**Figure 8:**
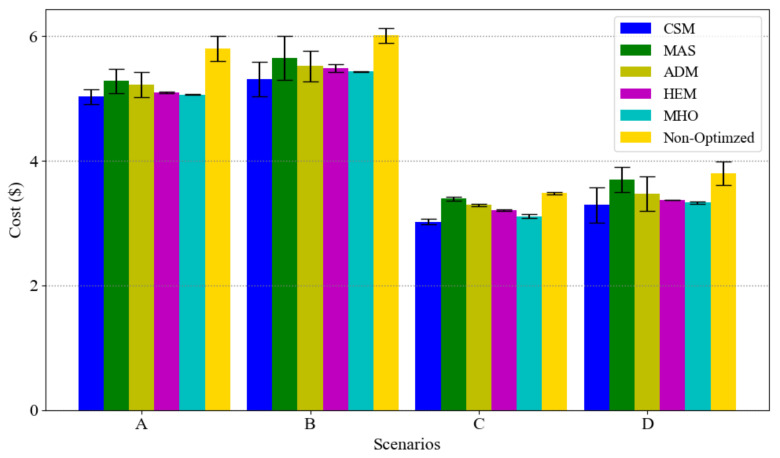
Total cost in a winter day on different scenarios with confidential interval.

**Figure 9:**
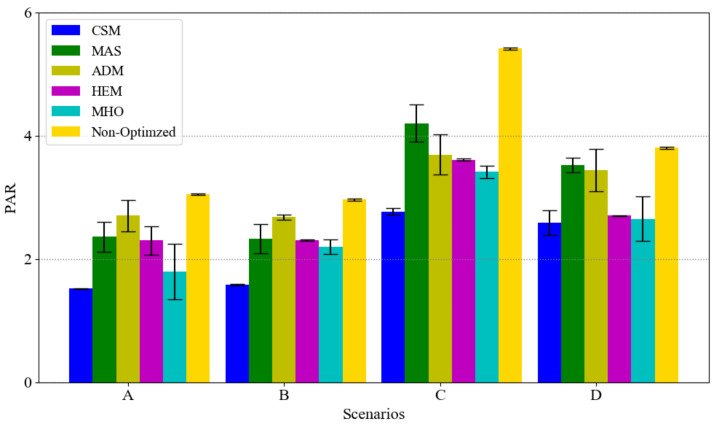
PAR on different scenarios with confidential interval.

To present the effect of load clustering and prioritization on our model, Figure 8 and Figure 9 present the results of different scenarios on the total cost and PAR, respectively. Note that, in each scenario, the total power demand is equal between the six approaches.

Based on Figure 8, our model has the lowest cost in all the scenarios considered. The appliances’ usage priority cause that elastic loads, with the high consumptions, are optimized first and then the prioritized deferrable loads optimized in next level. Moreover, considering essential loads usage as a lower bound in optimization model helps to reduce the total cost either.

Regarding to Figure 9, the PAR in our model is the minimum one and the reason why ADM has less PAR than MAS in scenarios C and D is due to the interruptible deferrable load assumption in ADM model (in scenario C and D number of deferrable loads are increased). Moreover, HEM and MHO has less PAR than ADM and MAS, because of their optimization models, GA and MILP. Also, it shows that the appliance usage priority and clustering have positive effects on finding proper time slots for the appliances consumption especially for the elastic load with high demand. Finally, we can assert that we reduced the cost almost 8%, 6%, 5% and 3%, and reduced PAR almost 34%, 33%, 24% and 17% more than MAS, ADM, HEM and MHO respectively.

## 4. Conclusions

In this paper we have presented a multi-objective demand management approach using appliance clustering and prioritization, and keeping the customer’s thermal comfort in ASHRAE standard range. Customer’s comfort is considered in many aspects, prioritizing appliances for the sequential optimization (the one optimized first will completely satisfy all its constraints), customer’s comfort on thermal load, EV’s state of charging, and deferrable loads’ non-interruptible usage on selected permissible time interval.

Our main goals are to flat the household demand and effectively reduce the customer’s cost while increasing customer comfort via their elastic and deferrable loads. In this work, we compared our light-weight model with other demand management methods, which have similarities in prioritization, clustering, PAR and cost management. Our results represent that we smoothed the load profile and reduced PAR almost 45% more than the non-optimized case, decreased the electricity bill almost 13%, keep the room’s temperature in ASHRAE standard range and charge EV more than the customer’s desired amount.

## Figures and Tables

**Table 1:** Categorizing the reviewed papers.

Paper	Objectives			Techniques	Real-Data	Load Category	Load Prioritization
	Cost	Appliance Usage	Thermal Comfort				
[7]	✔	✔		NLP	✔	✔	✔
[8]	✔	✔		ColorPower algorithm		✔	✔
[9]	✔	✔		NLP		✔	✔
[10]	✔	✔		GA		✔	
[11]	✔	✔		LP			✔
[12]	✔	✔		Minority Game	✔		✔
[13]	✔			NLP			
[14]	✔			LP			
[15]	✔			LP			✔
[16]	✔			LP			
[17]	✔		✔	LP			✔
[18]	✔		✔	Non-convex			
[19]			✔	DLC	✔		✔
[20]	✔		✔	MILP	✔		
[21]			✔	ML	✔		
[22]			✔	PSO			
[23]	✔		✔	LP	✔		
[24]	✔		✔	NLP			
[25]	✔	✔		LP	✔		
[26]	✔			LP			✔
[27]	✔	✔		MINP			
[28]	✔	✔		MILP	✔		✔
[29]	✔	✔		LP			✔
[30]	✔	✔		Problem-Solving approach	✔	✔	✔
[31]	✔			BPSO			✔

**Table 2:** Types of appliances.

Appliance	Load Type	Energy (kW/day)
Heater	Elastic	25.43
EV	Elastic	26
Freezer	Deferrable	2.07
Washing Machine	Deferrable	1.96
Cloth Dryer	Deferrable	2.47
Dish Washer	Deferrable	1.44
Refrigerator	Essential	3.65
Coffee Maker	Essential	0.19
TV	Essential	2.57
Light	Essential	0.41
Stove	Essential	0.61
PC	Essential	3.93

**Table 3:** Load profile scenarios.

Scenarios	Load Type						Total Energy (kW/day)
	Essential		Elastic		Deferrable		
	Qty.	Pct.	Qty.	Pct.	Qty.	Pct.	
Scenario A	6	17.7%	2	80.1%	1	2.2%	64.23
Scenario B	5	16.9%	2	77.8%	2	5.3%	66.11
Scenario C	4	18.5%	1	66.5%	3	15%	39.11
Scenario D	3	16.6%	1	63.6%	4	19.8%	40

**Table 4:** Results comparison.

Approach	PPD (%)	$T_{min}\;\left(℃\right)$	$T_{max}\;\left(℃\right)$
CSM	11.68	22	24.35
MAS	13.83	21.88	23.99
ADM	13.37	21.89	24.11
HEM	14.27	22	23.46
MHO	13.99	21.83	23.66

## Data Availability

Data sharing not applicable.

## Abbreviations

**Acronym**	**Description**
AC	Air Conditioner
AHP	Analytic Hierarchy Process
ADM	Autonomous Demand-side Management
BPSO	Binary Particle Swarm Optimization
CSM	Clustered Sequential Management
DER	Distributed Energy Resource
DR	Demand Response
DSM	Demand Side Management
EMS	Energy Management System
GA	Genetic Algorithm
GHG	Greenhouse Gas
HEM	Household Energy Management
HEMS	Home Energy Management System
HVAC	Heating Ventilation and Air Conditioner
IIoT	Industrial IoT
IoT	Internet of Things
LP	Linear Programming
MAS	Multi-class Appliances Scheduling
MHO	Multi-objective Household appliance Optimization
MILP	Mixed Integer Linear Programming
MINP	Mixed Integer Non-linear Programming
ML	Machine Learning
NAN	Neighborhood-Area Network
NLP	Non-linear Programming
OEB	Ontario Energy Board
PAR	Peak-to-Average Ratio
PMV	Predicted Mean Vote
PPD	Predicted Percentage Dissatisfaction
PSO	Particle Swarm Optimization
TA	Transformer Agent
ToU	Time of Use
